# Surveying Self-Medication Practices and Attitudes Toward Antibiotic Use Among Adults in Primary Care

**DOI:** 10.7759/cureus.96114

**Published:** 2025-11-04

**Authors:** Turki M Alanzi, Nouf Alanzi, Hend Muharraq, Karmah Alrumman, Norah Bin Hamad, Ghaidaa Alsayed, Abdullah Alhumaidan, Lulu Alharbi, Mohammed Rakan, Rafah Oudah, Rawan Alqarni, Yousef Alsuwayyid, Awatif Hakami, Shroug Essam Aldden

**Affiliations:** 1 Health Information Management and Technology, Imam Abdulrahman Bin Faisal University, Dammam, SAU; 2 College of Applied Medical Sciences, King Saud University, Riyadh, SAU; 3 College of Pharmacy, Jazan University, Jazan, SAU; 4 Microbiology Department, Alfaisal University, Riyadh, SAU; 5 College of Medicine, King Saud University, Riyadh, SAU; 6 Faculty of Medicine, King Abdulaziz University, Jeddah, SAU; 7 College of Medicine, Prince Sattam bin Abdulaziz University, Al-Kharj, SAU; 8 Department of Clinical Pharmacy, College of Pharmacy, Qassim University, Unaizah, SAU; 9 College of Pharmacy, King Saud University, Riyadh, SAU; 10 College of Pharmacy, University of Hafar Al-Batin, Hafar Al-Batin, SAU; 11 Pharmacy Department, Al Hammadi Hospital, Riyadh, SAU; 12 Faculty of Medicine, Ibn Sina University, Khartoum, SDN

**Keywords:** antibiotics, antimicrobial resistance, primary care, public health, saudi arabia, self-medication

## Abstract

Background: Antibiotic self-medication (ASM) is a significant global health concern contributing to antimicrobial resistance (AMR), particularly in low- and middle-income countries, including Saudi Arabia. Despite regulatory controls, inappropriate antibiotic use persists in primary care settings.

Aim: This study aimed to assess the prevalence, sources, and behavioral patterns of ASM and to evaluate knowledge, awareness, and attitudes toward antibiotic use among adults attending primary care clinics in Saudi Arabia.

Methods: A cross-sectional survey was conducted with 421 adult participants selected through random sampling from various primary care centers in Saudi Arabia: Family and Community Medicine Center, Dammam, and Imam Abdulrahman Bin Faisal University PHC, Dammam. A structured, self-administered questionnaire captured demographic data, antibiotic use frequency, sources, knowledge, awareness of AMR, and attitudes. Data were analyzed using IBM SPSS Statistics, applying descriptive statistics and analysis of variance (ANOVA).

Results: Of the participants, 276 (65.6%) had used antibiotics in the past year. Notably, 207 (49.2%) reported buying antibiotics without prescriptions, and 189 (44.9%) used leftovers for common illnesses. Educational level was significantly associated with usage frequency (p < 0.0001). Knowledge scores showed no significant demographic differences, while AMR awareness increased significantly with age (p < 0.0001). Furthermore, 226 (53.7%) took antibiotics for colds or sore throats, and 214 (50.8%) stopped treatment once they felt better.

Conclusion: Widespread ASM and persistent misconceptions highlight the urgent need for targeted behavioral and regulatory interventions in primary care.

## Introduction

The inappropriate use of antibiotics has emerged as a critical global health concern, with self-medication practices playing a significant role in accelerating antimicrobial resistance (AMR) [1-3]. The World Health Organization (WHO) identifies AMR as one of the top ten global public health threats, stressing that irrational and unregulated consumption of antibiotics undermines their therapeutic efficacy, increases healthcare costs, and leads to prolonged hospital stays and higher mortality rates [4,5]. Among the various factors contributing to this crisis, self-medication, which is defined as the use of drugs to treat self-diagnosed conditions without medical supervision, poses a particularly serious challenge, especially when it involves antibiotics [6].

In many countries, including Saudi Arabia, the misuse of antibiotics through self-medication is widespread. A recent review [7] of 71 studies with 63,251 participants revealed a global antibiotic self-medication (ASM) prevalence of 43%. Prevalence was highest in sub-Saharan Africa (55.2%) and among students (62.1%). Key reasons for ASM included antibiotic knowledge, previous successful use, and perceiving illness as minor [7]. Despite existing regulations requiring prescriptions for antibiotic dispensation, community pharmacies often continue to serve as informal points of access. Adults frequently resort to self-medicating with antibiotics for common ailments such as colds, sore throats, and fevers, driven by convenience, previous successful experiences, time constraints, or a lack of trust in healthcare services [7-11].

Similar results were observed in Saudi Arabia [12], where a recent study observed that 34% prevalence of using antibiotics without a prescription, despite 81.3% being aware of potential health risks. Amoxicillin/clavulanic acid (45.1%) was the most used. Tonsillitis (76.7%) and prior experience (52.1%) were key reasons, with previous doctor prescriptions (36.6%) being the main source. These behaviors are often reinforced by a limited understanding of antibiotic indications, confusion between bacterial and viral infections, and misconceptions about the effectiveness of antibiotics in treating non-bacterial illnesses. A recent study [13] has highlighted that multidimensional poverty in low- and middle-income countries (LMICs) significantly contributes to the rise of AMR by limiting access to healthcare, weakening regulatory systems, reducing health literacy, and encouraging self-medication without appropriate prescriptions. Although the prevalence of ASM varied across the reviewed studies, it consistently remained at high levels. A wide range of antibiotics, spanning multiple classes such as cell wall synthesis inhibitors, protein synthesis inhibitors, and DNA synthesis inhibitors, were frequently used in self-medication practices. Common cultural factors influencing ASM, as identified in the literature, included prior personal experiences with antibiotics and the broader cultural acceptance of self-directed antibiotic use.

Previous research has consistently documented a high frequency of antibiotic use among adults in primary care settings, much of which occurs without medical supervision. Several studies [1-3,7,12] have highlighted that antibiotic self-medication remains a widespread practice, particularly in low- and middle-income countries. ASM is a significant and persistent issue globally, with the reported prevalence among adults reaching as high as 43% in pooled global estimates [7]. These findings are mirrored regionally, such as in a cross-sectional study from Colombia, where 46% of adults reported ASM, most frequently for flu-like symptoms, including those associated with COVID-19 [14]. In Asia, prevalence varies but remains high in certain communities, as shown in a community study from Malaysia, which found that 13.3% of primary care attendees engaged in ASM [15]. Across these studies, students and younger adults are consistently identified as high-frequency users, with a meta-analysis indicating ASM rates of 62.1% among students versus 32.6% in the general public [7,14,15].

Understanding of antibiotics among adult populations continues to be inconsistent and often inadequate. A study in Vietnam [16] observed that, although those who self-medicated had somewhat higher knowledge scores about antibiotics, this did not translate to safer practices; many retained antibiotics at home and used leftovers for new respiratory complaints. Internationally, a study in Malawi [17] similarly revealed widespread misconceptions, with community members frequently unable to distinguish between bacterial and viral infections in the context of antibiotic use. Several studies, such as recent systematic reviews [7,12], identified previous personal experience with similar symptoms and earlier physician prescriptions as primary drivers for repeated ASM, emphasizing that knowledge alone may not change behaviors routinely.

Awareness of AMR is growing but remains superficial in scope. The Malaysian clinic study [15] noted that 70.6% of respondents recognized antibiotic overuse could lead to resistance; nonetheless, such understanding did not sufficiently discourage inappropriate use. The survey in Colombia found a weak correlation between AMR awareness and proper antibiotic consumption, with about half of the participants still using antibiotics for viral infections despite acknowledging resistance issues [14]. Regional disparities persist, with sub-Saharan African studies reporting gaps in both conceptual and practical knowledge regarding AMR, often due to limited access to public health information and inconsistent policy enforcement [18-21].

Attitudes toward antibiotic use reflect significant cultural, socioeconomic, and informational influences [22-24]. Negative or permissive attitudes are strongly associated with higher ASM rates [25-27]. For instance, the studies have demonstrated that individuals with poorer attitudes toward antibiotics, such as supporting the use of leftover antibiotics or advocating ASM for minor symptoms-were more likely to self-medicate, and this relationship was statistically significant [28,29]. Similarly, a systematic review found attitudes shaped not just by education but also by societal norms and health system accessibility, with many participants citing convenience, cost, and trust in informal sources (like pharmacists or previous prescriptions) as major motivators for ASM [7,12]. Notably, policies restricting over-the-counter antibiotic sales were only moderately effective, as leftover and shared antibiotics remained a common supply channel.

Thus, it can be observed that a high and sometimes growing frequency of antibiotic self-medication among adults, variable but often insufficient knowledge of appropriate use, limited depth of awareness about AMR, and predominantly permissive or uninformed attitudes toward antibiotic consumption. These challenges are compounded by socioeconomic factors and the pervasive influence of personal experience and informal advice. The evidence from diverse regions and study types points to the urgent need for multifaceted interventions focused not only on improving knowledge and regulation but also on shifting attitudes and personal beliefs about antibiotics and resistance in the primary care context.

Primary care settings are at the forefront of managing infectious diseases and offer an important platform for addressing these challenges. They serve as the initial point of contact for the public and play a pivotal role in promoting appropriate medication use. Assessing self-medication practices within these settings provides valuable insights into patient behaviors and helps identify critical areas for intervention. This study seeks to investigate the prevalence and patterns of self-medication with antibiotics and to examine the attitudes and beliefs that underpin these behaviors among adults in primary care settings across Saudi Arabia. The significance of this research lies in its potential to inform national efforts aligned with Saudi Arabia’s Vision 2030 health objectives, which emphasize preventive care, patient education, and improved healthcare delivery systems. 

The primary objective of this study was to determine the prevalence of self-medication with antibiotics (ASM) among adults attending primary care clinics in Saudi Arabia.

The secondary objectives were to 1) identify the most common sources through which antibiotics are obtained (e.g., prescription, leftover medication, over-the-counter purchase, or sharing); 2) describe behavioral patterns related to antibiotic self-medication, including the frequency of use, types of illnesses for which antibiotics are used, and adherence behaviors such as premature discontinuation; 3) evaluate participants’ knowledge about appropriate antibiotic use through a structured 10-item scale assessing understanding of antibiotic indications and risks; 4) assess awareness of AMR and its association with demographic variables such as age, gender, and education; and 5) examine attitudes toward antibiotic consumption, including tendencies to self-medicate, store leftover antibiotics, and comply with medical advice.

## Materials and methods

Study settings and participants

This cross-sectional study was conducted across various primary care clinics in Saudi Arabia: Family and Community Medicine Center, Dammam, and Imam Abdulrahman Bin Faisal University PHC, Dammam. The target population comprises adult individuals aged 18 years and above who are attending these clinics during the study period. The study focuses on evaluating self-medication practices with antibiotics and related attitudes and knowledge. Participants must be capable of providing informed consent and completing a self-administered questionnaire, either electronically or in person.

Sampling

A random sampling technique [30] was used to recruit participants from clinic attendees to minimize selection bias and ensure representativeness. The minimum sample size, calculated using Cochran's formula with a 95% confidence level and a 5% margin of error, is 385 participants. This estimate assumes a prevalence rate of 50% for maximum variability, thus ensuring sufficient power to detect statistically significant associations.

Questionnaire design

The data collection tool used in this study is a structured, self-administered questionnaire specifically developed to assess self-medication practices and attitudes toward antibiotic use among adult patients attending primary care clinics in Saudi Arabia (see Appendix). The questionnaire was designed based on an extensive review of relevant literature [7,12,31,32] and adapted from validated instruments previously used in similar studies [31,32]. It was constructed to capture comprehensive information across multiple dimensions, including participants’ demographic characteristics, patterns of antibiotic use, knowledge of antibiotics, awareness of antimicrobial resistance, and attitudes toward antibiotic consumption.

The first section of the questionnaire focuses on demographic information, capturing variables such as age, gender, health insurance status, education level, employment status, and whether any immediate family member works in the healthcare sector. These variables are essential for understanding how sociodemographic factors influence antibiotic use behaviors and perceptions. The second section explores the frequency and sources of antibiotic use. Participants are asked whether they have used antibiotics in the past year, how frequently, and where they obtained them. Response options include prescribed use, over-the-counter purchases, leftover medications, or antibiotics acquired from friends or family members. This section helps identify common sources and behaviors associated with antibiotic self-medication.

The third section assesses knowledge about antibiotics using a series of 10 statements rated on a five-point Likert scale ranging from strong disagreement to strong agreement. The items evaluate participants’ understanding of what antibiotics are, their appropriate use, potential side effects, and the risks of misuse. For example, statements such as “Antibiotics are not useful for viral infections like the common cold” and “It is not safe to share my prescribed antibiotics with a family member who has similar symptoms” are included to measure factual knowledge and dispel common misconceptions. The fourth section addresses awareness of antibiotic resistance. It includes both binary and Likert-scale questions to evaluate whether participants have heard of antimicrobial resistance and where they obtained such information (e.g., healthcare providers, media, internet). It also assesses their understanding of key concepts, such as the consequences of incomplete antibiotic courses and the broader public health implications of resistance. This section is important for gauging public awareness and identifying educational needs. The final section examines participants’ attitudes and behaviors regarding antibiotic use. It consists mainly of yes/no questions that explore practical habits and beliefs, such as whether individuals typically complete prescribed courses, use leftover antibiotics, purchase antibiotics without a prescription, or feel pressured to take antibiotics unnecessarily. This section provides insight into personal and cultural tendencies that may contribute to misuse and inform future behavior-change interventions.

To ensure linguistic clarity and cultural appropriateness, the questionnaire was translated from English to Arabic and reviewed by two bilingual academic experts. Their feedback was incorporated to refine grammar, enhance clarity, and improve cultural relevance. A pilot study was then conducted with 15 randomly selected participants to test the questionnaire’s reliability and ease of comprehension. The internal consistency of the survey instrument was evaluated using Cronbach’s alpha, with results exceeding the accepted threshold of 0.7 [33], confirming the tool’s reliability for broader data collection.

Data collection

Data were collected over a five-week period (July 1, 2025, to August 8, 2025) through both electronic and paper-based formats to maximize accessibility and response rates. Participants were recruited via email invitations and in-person approaches at participating primary care clinics. Each invitation included details about the study purpose, procedures, and assurances of confidentiality.

Data analysis

Collected data were entered into a secure, password-protected database and analyzed using IBM SPSS Statistics version 30 (released 2024, IBM Corp., Armonk, NY). Descriptive statistics (frequencies, percentages, means, and standard deviations) were used to summarize participant demographics and response patterns. Inferential statistics were employed to examine associations between self-medication practices and demographic or attitudinal variables. Specifically, ANOVA analyses were conducted, with statistical significance set at p < 0.05.

Ethical considerations

This study was conducted in strict adherence to ethical research principles to ensure the rights, safety, and well-being of all participants. Ethical approval was obtained from the appropriate institutional review board prior to data collection. Participants were provided with detailed information about the purpose, procedures, and voluntary nature of the study through a consent form attached to the online survey. It was clearly stated that participation was entirely voluntary and that respondents could withdraw at any time without any consequences.

## Results

The demographic profile of the 421 study participants (see Table 1) reveals a nearly equal distribution between males and females. The age distribution shows a relatively balanced representation, with the largest age group being those above 51 years, followed by participants aged 41-50 and 18-30, and 31-40. Educational attainment varies, with the majority having at least a high school education. Only a small proportion had advanced degrees, including master's and doctoral degrees. Employment status indicates that the largest group was homemakers, followed by the unemployed, employed individuals, disabled, and retired participants. About one-third of the participants are recruited from the Family and Community Medicine Center, and the remaining from Imam Abdulrahman Bin Faisal University PHC. Notably, only 42 (10%) reported having at least one family member working in a health-related field, highlighting limited direct exposure to healthcare knowledge within households.

**Table 1:** Participants' demographics

Variables	Sub-variable	N	Relative frequency
Gender	Male	214	50.8%
	Female	207	49.2%
Age (in years)	18-30	106	25.2%
	31-40	82	19.5%
	41-50	109	25.9%
	> 51	124	29.5%
Education	Less than High School / Uneducated	67	15.9%
	High School	97	23.0%
	College/ Diploma	94	22.3%
	Bachelor's Degree	92	21.9%
	Master’s Degree	48	11.4%
	Doctoral Degree	23	5.5%
Employment	Employed	83	19.7%
	Unemployed	96	22.8%
	Retired	46	10.9%
	Homemaker	149	35.4%
	Disabled	47	11.2%
Centers	Family and Community Medicine Center	314	74.6%
	Imam Abdulrahman Bin Faisal University PHC	107	25.4%
At least one member of your family (parents, children, husband/wife) works in a health-related field?	Yes	42	10.0%
	No	379	90.0%

Out of the 421 participants, 276 (65.6%) individuals reported using antibiotics in the past year, while 145 (34.4%) indicated that they had not. This suggests a relatively high frequency of antibiotic use among adults in the primary care setting.

To assess differences in the frequency of antibiotic use among participant groups, a one-way ANOVA was conducted (see Table 2). Frequency responses were numerically coded as follows: “1-2 times” = 1, “3-5 times” = 3, and “>5 times” = 5. This ordinal transformation allowed the calculation of means and variances across demographic categories. The analysis, based on the 276 participants who reported using antibiotics in the past year, revealed no statistically significant differences in frequency of use based on gender (p = 0.6066), age groups (p = 0.4152), or having a family member in a healthcare field (p = 0.3897), indicating relatively similar usage patterns within these groups. However, a highly significant difference was found among participants with different education levels (p < 0.0001). Participants with lower educational attainment, particularly those with less than a high school education, had a much higher mean frequency of antibiotic use (mean = 4.59) compared to those with advanced degrees, such as master’s (mean = 1.73) and doctoral (mean = 1.43). This finding suggests that education level is a critical factor influencing antibiotic usage, with higher education associated with more restrained and possibly more appropriate use.

**Table 2:** ANOVA of the frequency of antibiotic use among the participant groups (N = 276) * Statistically significant difference (p < 0.05)

Variables	Sub-variables	N	Mean	Variance	P-value
Gender	Male	134	2.72	2.42	0.6066
	Female	142	2.62	2.44	
Age (in years)	18-30	71	2.77	2.69	0.4152
	31-40	57	2.58	2.39	
	41-50	62	2.42	2.15	
	> 51	86	2.81	2.41	
Education	Less than high school / uneducated	49	4.59	0.66	< 0.0001*
	High school	62	2.45	2.12	
	College/diploma	60	2.77	2.45	
	Bachelor's degree	58	2.00	1.02	
	Master’s degree	33	1.73	0.95	
	Doctoral degree	14	1.43	0.73	
Does any member of the family work in a healthcare-related field?	Yes	42	2.86	2.42	0.3897
	No	234	2.63	2.42	

The findings on the methods of obtaining antibiotics indicate that the most common source was through a doctor's prescription, reported by 73.6% of participants, with a relatively low mean frequency of use (mean = 2.4, rank = 2). This suggests that while most individuals obtain antibiotics appropriately, alternative and potentially inappropriate sources are also prevalent. Notably, 265 (62.9%) reported using leftover antibiotics from previous prescriptions, although this group had the lowest mean frequency (1.8), but ranked third, implying sporadic but widespread practice. Over-the-counter (OTC) purchase without a prescription was reported by 132 (31.4%) participants and had the highest mean frequency (4.4), reflecting concerning patterns of frequent self-medication. Lastly, 110 (26.1%) obtained antibiotics from family or friends, with a moderate mean frequency (2.8) and the highest rank (5), highlighting another informal and less regulated channel of access. These findings point to significant reliance on non-prescribed antibiotic sources, which could contribute to inappropriate usage and resistance development.

The ANOVA analysis of participants’ knowledge about antibiotic use (see Table 3) shows no statistically significant differences across any of the examined demographic groups. Gender-wise, females had a slightly higher mean knowledge score (2.86) than males (2.79), although the difference was not significant (p = 0.0598). Age-based differences were minimal, with mean scores ranging narrowly from 2.81 to 2.85 across all age groups (p = 0.8367). Similarly, educational level did not significantly influence knowledge scores (p = 0.9278), with all education groups showing comparable means around 2.8, including both the least and most educated participants. In addition, having a family member working in a healthcare-related field did not significantly affect knowledge levels (p = 0.6441).

**Table 3:** ANOVA of the knowledge of antibiotic use among the participant groups (N = 421) * Statistically significant difference (p < 0.05)

Variables	Sub-variables	N	Mean	Variance	P-value
Gender	Male	214	2.79	0.15	0.0598
	Female	207	2.86	0.13	
Age (in years)	18-30	106	2.82	0.13	0.8367
	31-40	82	2.82	0.17	
	41-50	109	2.85	0.15	
	> 51	124	2.81	0.13	
Education	Less than high school / uneducated	67	2.84	0.14	0.9278
	High school	97	2.84	0.17	
	College/diploma	94	2.81	0.16	
	Bachelor's degree	92	2.82	0.12	
	Master’s degree	48	2.83	0.11	
	Doctoral degree	23	2.75	0.14	
Does a member of the family work in a healthcare-related field?	Yes	42	2.80	0.21	0.6441
	No	379	2.83	0.13	

The ANOVA results for the awareness of antibiotic resistance among participants (see Table 4) reveal significant differences based on age and education level. Although no significant gender-based difference was observed (p = 0.864), and having a family member in a healthcare-related field also showed no significant influence (p = 0.6276), age showed a clear and statistically significant impact (p < 0.0001). Awareness scores increased with age, from a mean of 5.19 in the 18-30 group to 12.03 in those over 51, suggesting that older individuals are substantially more aware of antibiotic resistance issues. Educational level also had a statistically significant impact (p = 0.0461), with participants holding college diplomas or master’s degrees demonstrating slightly higher awareness compared to those with less or more formal education. Interestingly, doctoral degree holders did not show the highest mean awareness, indicating possible variation in exposure to public health education regardless of academic level.

**Table 4:** ANOVA of the awareness of antibiotic resistance among the participant groups (N = 421) * Statistically significant difference (p < 0.05)

Variables	Sub-variables	N	Mean	Variance	P-value
Gender	Male	214	8.83	7.04	0.864
	Female	207	8.88	7.62	
Age (in years)	18-30	106	5.19	0.54	< 0.0001*
	31-40	82	7.69	0.38	
	41-50	109	9.68	0.42	
	> 51	124	12.03	0.62	
Education	Less than high school/uneducated	67	8.34	9.05	0.0461*
	High school	97	9.01	6.05	
	College/diploma	94	9.40	6.36	
	Bachelor's degree	92	8.30	6.98	
	Master’s degree	48	9.18	9.01	
	Doctoral degree	23	8.99	7.26	
Does any member of the family work in a healthcare-related field?	Yes	42	9.05	7.13	0.6276
	No	379	8.83	7.34	

The findings on the participants’ attitudes toward antibiotic use and consumption (see Table 5) reveal a mix of appropriate and inappropriate practices. A considerable proportion (226; 53.7%) reported taking antibiotics for a cold or sore throat, and 213 (50.6%) for fever, both typically viral conditions where antibiotics are not usually indicated. Half of the respondents (214; 50.8%) admitted to stopping antibiotics when they began feeling better, which can contribute to antibiotic resistance. Worryingly, only 199 (47.3%) stated that they take antibiotics only when prescribed by a doctor, while the rest may be engaging in self-medication. Nearly one-third (129; 30.6%) keep leftover antibiotics for future use, and 189 (44.9%) use them without medical consultation during common illnesses. In addition, 207 (49.2%) reported purchasing antibiotics without a prescription, and 210 (49.9%) had started antibiotic therapy after only a phone consultation. These behaviors suggest widespread access and usage without proper medical oversight. Moreover, 261 (62%) participants incorrectly believe antibiotics are always necessary to treat infections, and 209 (49.6%) reported feeling pressured by their doctor to take antibiotics even when unsure of the need.

**Table 5:** Attitudes of the participants toward antibiotic use and consumption

Items	Yes	No
Do you usually take antibiotics for a cold or sore throat?	226 (53.7%)	195 (46.3%)
Do you usually take antibiotics for fever?	213 (50.6%)	208 (49.4%)
Do you usually stop taking antibiotics when you start feeling better?	214 (50.8%)	207 (49.2%)
Do you take antibiotics only when prescribed by a doctor?	199 (47.3%)	222 (52.7%)
Do you keep leftover antibiotics at home because they might be useful in the future?	129 (30.6%)	292 (69.4%)
Do you use leftover antibiotics when you have a cold, sore throat, or flu without consulting your doctor?	189 (44.9%)	232 (55.1%)
Do you buy antibiotics without a medical prescription?	207 (49.2%)	214 (50.8%)
Have you ever started an antibiotic therapy after a simple doctor call, without a proper medical examination?	210 (49.9%)	211 (50.1%)
I believe antibiotics are always necessary for treating infections.	261 (62%)	160 (38%)
I feel pressured by my doctor to take antibiotics even if I don't think I need them.	209 (49.6%)	212 (50.4%)

## Discussion

The findings of this study confirm that ASM remains a prevalent and pressing issue in primary care settings in Saudi Arabia, aligning with global trends observed in LMICs. Despite regulatory restrictions, 49.2% of the participants reported buying antibiotics without a prescription, closely mirroring global ASM rates of 43% as reported in a recent meta-analysis [7]. This reinforces prior findings from regional studies [12], which documented widespread non-prescription antibiotic use despite awareness of associated health risks. The high rate of use of antibiotics for viral conditions such as colds (226; 53.7%) and fevers (213; 50.6%) also aligns with patterns seen in studies from Colombia and Malaysia [14,15], where antibiotics were frequently used to treat flu-like symptoms. These findings suggest a disconnect between knowledge and practice, underscoring the need for behaviorally focused public health interventions.

Interestingly, although knowledge scores were statistically similar across demographic groups, there remained a widespread belief in misconceptions; 261 (62%) participants believed that antibiotics are always necessary to treat infections. This supports earlier findings from Vietnam [16] and Malawi [17], where higher knowledge did not necessarily lead to better practices. This paradox highlights that information alone is insufficient to change behavior; attitudes, past experiences, and cultural norms play equally critical roles. Furthermore, 214 (50.8%) respondents admitted to stopping antibiotics when symptoms subsided, an issue consistently reported in the literature [14,15], contributing to the persistence of AMR despite rising awareness.

Education level emerged as a critical differentiator in antibiotic usage patterns. Participants with lower educational attainment reported significantly higher frequencies of antibiotic use (p < 0.0001), reinforcing the claim from [7] that inadequate education is linked to poor antibiotic stewardship. However, knowledge scores did not differ significantly by education, suggesting that deeper attitudinal and experiential factors may be more influential in guiding antibiotic behavior than knowledge alone [34]. This insight is consistent with the broader body of research, which posits that culturally embedded behaviors, perceived illness severity, and past positive outcomes with antibiotics often override formal knowledge in decision-making [22-27]. Although education level was significantly associated with antibiotic-use frequency, it did not predict differences in knowledge scores. This finding suggests that formal education may shape behavioral restraint more than factual knowledge about antibiotics. Prior research indicates that antibiotic-related understanding depends less on academic achievement and more on targeted health literacy and experiential learning [35-37]. In Saudi Arabia, widespread access to mass media health campaigns and culturally embedded beliefs about antibiotics may also homogenize knowledge levels across educational groups. However, individuals with higher education may demonstrate more cautious antibiotic use behaviors due to greater risk perception and access to professional healthcare advice. These results underscore that education alone is insufficient for ensuring rational antibiotic use; behavioral interventions must complement informational campaigns to bridge the gap between knowledge and practice.

A notable contribution of this study is its emphasis on age-related variations in AMR awareness. Participants above 51 demonstrated significantly higher awareness levels (mean = 12.03) compared to younger groups, suggesting that exposure and personal health history may enhance understanding of AMR over time. However, younger adults, particularly those under 30, remain a critical group for targeted interventions, as they displayed the lowest awareness (mean = 5.19), reflecting findings from earlier studies [7,14,15] that identified students and young adults as high-risk ASM users.

Finally, the finding that nearly half of participants initiated antibiotics after a phone call without examination (210, 49.9%) or kept leftovers for future use (129, 30.6%) reflects a lack of enforcement around antibiotic dispensing policies and a strong reliance on informal or convenience-based decision-making [38,39]. This matches previous literature indicating that even where over-the-counter sales are restricted, informal networks and leftover medications remain potent sources of ASM [7,12].

Study limitations

This study is subject to several limitations. First, the use of self-reported questionnaires may introduce recall or social desirability bias. Second, although random sampling was applied, the sample is restricted to clinic attendees and may not reflect the broader Saudi population. Third, the cross-sectional design limits causal inferences. Additionally, the questionnaire, though validated, may not have captured all dimensions of cultural or economic influences affecting ASM. Fourth, while univariate ANOVAs were employed to assess demographic differences across knowledge, awareness, and behavioral variables, this approach does not account for possible correlations among dependent variables. A multivariate analysis (e.g., MANOVA) could offer a more integrated interpretation of these interrelationships. Finally, while statistical significance was tested, the reliance on ANOVA may overlook complex interactions among variables best explored through multivariate or longitudinal analyses.

Contributions

This study contributes theoretically by reinforcing the limited influence of knowledge alone on self-medication behavior, underscoring the significance of health behavior models that account for attitudes and cultural norms. Practically, it highlights the urgent need for targeted public health interventions focused not only on education but also on attitude change, especially among younger adults and those with limited formal education. Additionally, the results support policy development on tighter regulation of antibiotic sales and more robust enforcement mechanisms. Primary care clinics are reaffirmed as crucial intervention points for antibiotic stewardship and patient education in Saudi Arabia.

Recommendations

Based on the findings, several recommendations are proposed. First, national health campaigns should move beyond information dissemination to target behavioral change, emphasizing risks associated with ASM and benefits of completing antibiotic courses. These should be tailored especially for younger adults and individuals with lower education levels. Second, stricter enforcement of prescription-only policies for antibiotics is necessary, with penalties for non-compliant pharmacies. Third, healthcare providers should be trained to communicate AMR risks effectively and avoid unnecessary antibiotic prescriptions, particularly in virtual or telephonic consultations. Fourth, primary care clinics should incorporate antibiotic education sessions during routine visits. Finally, community engagement initiatives-through schools, workplaces, and social media-should focus on altering cultural perceptions and normalizing appropriate antibiotic use. Future research should explore qualitative insights into patient motivations and evaluate the long-term impact of multifaceted interventions combining policy, education, and health system reform.

## Conclusions

This study provides critical insights into the prevalence and patterns of ASM among adults in Saudi Arabian primary care settings. Despite moderate levels of knowledge and awareness about antibiotics and AMR, a substantial proportion of participants reported inappropriate antibiotic use behaviors, including purchasing antibiotics without prescriptions, using leftover medications, and discontinuing therapy prematurely. These practices, compounded by cultural norms and limited regulatory enforcement, pose significant risks for AMR development. Notably, education level was a key determinant of antibiotic usage frequency, but not necessarily of knowledge or awareness, emphasizing the need for targeted interventions that address behavior and attitudes in addition to informational gaps. Older adults demonstrated greater awareness of AMR, whereas younger adults, who are also high-frequency ASM users, require focused engagement. To curb the rise of AMR, a multifaceted approach involving public education, stricter policy enforcement, and culturally sensitive behavior-change strategies is essential. Primary care clinics represent a strategic platform for implementing such interventions and promoting rational antibiotic use.

## Appendices

**Table 6:** Survey questionnaire: self-medication practices and attitudes toward antibiotic use

Section	Question/statement	Response options
Demographic data	Age	__________
	Gender	Male / Female
	Do you have health insurance?	Yes / No
	Highest level of education	Less than High School / High School / College-Diploma / Bachelor’s / Master’s / Doctoral
	Employment status	Employed / Unemployed / Retired / Homemaker / Disabled
	Family member in a health-related field?	Yes / No
Frequency of use	Have you used antibiotics in the last year?	Yes / No
	If yes, how many times?	1–2 / 3–5 / > 5
	Where did you obtain the antibiotics?	Doctor / OTC / Leftovers / Family or Friend
Knowledge about antibiotics	Penicillin or Amoxicillin are antibiotics.	1 2 3 4 5
	Aspirin is not an antibiotic.	1 2 3 4 5
	Paracetamol is not an antibiotic.	1 2 3 4 5
	Antibiotics are useful for bacterial infections.	1 2 3 4 5
	Antibiotics are not useful for viral infections.	1 2 3 4 5
	Antibiotics are not indicated for pain/inflammation.	1 2 3 4 5
	Antibiotics can kill 'good bacteria'.	1 2 3 4 5
	Antibiotics can cause secondary infections.	1 2 3 4 5
	Antibiotics can cause allergic reactions.	1 2 3 4 5
	It is not safe to share prescribed antibiotics.	1 2 3 4 5
Awareness about antibiotic resistance	Have you ever heard about antibiotic resistance?	Yes / No
	If yes, where?	Doctor / Healthcare Provider / TV / Newspaper / Internet / Other: ____
	Antibiotic resistance is when bacteria lose sensitivity.	1 2 3 4 5
	Misuse can lead to resistance.	1 2 3 4 5
	The full course of antibiotics must be completed.	1 2 3 4 5
	Antibiotic resistance is a major public health problem.	1 2 3 4 5
	Resistance can affect anyone.	1 2 3 4 5
Attitudes regarding antibiotic consumption	Do you usually take antibiotics for a cold/sore throat?	Yes / No
	Do you usually take antibiotics for a fever?	Yes / No
	Do you stop taking antibiotics when you feel better?	Yes / No
	Do you take antibiotics only when prescribed?	Yes / No
	Do you keep leftover antibiotics at home?	Yes / No
	Do you use leftover antibiotics without a doctor?	Yes / No
	Do you buy antibiotics without a prescription?	Yes / No
	Started antibiotics after only a phone call?	Yes / No
	I believe antibiotics are always necessary.	Yes / No
	I feel pressured by the doctor to take antibiotics.	Yes / No
